# Development of a community-based network to promote smoking cessation among female smokers in Hong Kong

**DOI:** 10.1186/s12889-017-4213-z

**Published:** 2017-04-11

**Authors:** William H. C. Li, Sophia S. C. Chan, Zoe S. F. Wan, M. P. Wang, K. Y. Ho, T. H. LAM

**Affiliations:** 1 School of Nursing, The University of Hong Kong, 4/F, William MW Mong Block, No 21 Sassoon Road, Pokfulam, Hong Kong; 2 School of Public Health, The University of Hong Kong, G/F, Patrick Manson Building (North Wing), No 7 Sassoon Road, Pokfulam, Hong Kong

**Keywords:** Chinese, Female smokers, Gender-specific, Community-based network, Smoking cessation services

## Abstract

**Background:**

There is a need for population-based smoking cessation interventions targeting female smokers in Hong Kong. This study describes the development of a community-based network to promote smoking cessation among female smokers in Hong Kong.

**Methods:**

Local women’s organizations collaborated to launch a project to provide gender-specific smoking cessation services. In the first phase of the project, the Women Against Tobacco Taskforce (WATT) was created. In the second phase, a smoking cessation training curriculum was developed and female volunteers were trained. The third and final phase included the provision of gender-specific smoking cessation counseling services in Hong Kong.

**Results:**

A need assessment survey with 623 workers and volunteers of WATT members was carried out to develop a gender-specific smoking cessation training curriculum. A 1-day training workshop to 28 WATT affiliates who provided brief cessation counseling in the community was organized. Fourteen organizations (69 service units) agreed to form a network by joining WATT to promote smoking cessation and increase awareness of the specific health risks among female smokers.

**Conclusions:**

The community-based network to promote smoking cessation was effective in helping female smokers to quit smoking or reduce their cigarette consumption. The results also suggest that this community model of promoting gender-specific smoking cessation services is feasible.

**Trial registration:**

Clinicaltrials.gov ID NCT02968199 (Retrospectively registered on November 16, 2016).

**Electronic supplementary material:**

The online version of this article (doi:10.1186/s12889-017-4213-z) contains supplementary material, which is available to authorized users.

## Background

Although the prevalence of smoking has declined significantly over the last 50 years, the morbidity and mortality from smoking-related diseases, particularly among women, have risen greatly [1, 2]. There is strong evidence that smoking has detrimental effects on almost every organ of our body. Women are as likely as men to die from the diseases caused by smoking, including lung cancer, chronic obstructive pulmonary disease, or cardiovascular diseases [2, 3].

There is growing concern about the use of tobacco among women [4, 5]. The rates of smoking among women have increased dramatically since the first US Surgeon General’s report in 1964 [2], and it has been predicted that 20% of women worldwide will be smokers by 2025, compared with 12% in 2010 [6]. The daily cigarette smoking prevalence in Hong Kong has dropped from 23.3% in 1982 to 10.5% in 2015 [7]. Moreover, in 2015, approximately 3.5% of the female population in Hong Kong were daily smokers with their average consumption of 11 cigarettes per day [7]. A previous study found that the risk of death from smoking was the same for Chinese women and men in Hong Kong, and half of all deaths among smokers aged 65 years or older were caused by smoking [8]. Despite the overall low prevalence of smoking, the 103,000 female daily smokers in Hong Kong cannot be overlooked [7]. Of these female smokers, 62.4% have not attempted or do not want to quit, while 30.8% have tried but failed to quit smoking [7]. Although women are much less likely to smoke than men, the number of female smokers has risen by 83.6% since 1990, with a significant increase from 56,100 to 103,000 [7].

Nearly all female smokers (98.4%) in Hong Kong reported that they had not attended smoking cessation services provided by the government and other organizations, of which only 6.7% indicated that they would like to attend [9], demonstrating the need for population-based smoking cessation interventions targeting female smokers in Hong Kong. In response to the increased number of female smokers and their low usage rates of smoking cessation services, the first smoking cessation hotline for female smokers in Hong Kong was established in 2006 by the University of Hong Kong. An evaluation study was conducted to evaluate the effectiveness of this gender-specific smoking cessation hotline for female smokers [10]. The study provides some evidence for the effectiveness of the hotline in helping female smokers to quit or reduce smoking. Nevertheless, the study [10] did not report details on how this gender-specific hotline was established, especially the way to form a coalition and train people in smoking cessation services, and successfully launched the counseling services. The aim of this study was to describe the development of a community-based network to promote smoking cessation among female smokers in Hong Kong.

## Methods

This project, including the procedure to obtain informed consent and phase III, which evaluated the gender-specific smoking cessation counseling service from November 2006 to March 2012, was approved by the Institutional Review Board of the University of Hong Kong and Hospital Authority Hong Kong West Cluster (reference UW 06–323 T/1348). Eligible subjects were invited to participate in the study after being informed of its purpose. They were informed that their participation was voluntary and without prejudice, and given the opportunity to refuse to participate. The tailor-made intervention provided a flexible approach and schedule for counseling. Subjects selected either in-person or telephone counseling at baseline. Written consent was obtained from those subjects electing in-person counseling at baseline. Verbal consent was obtained from those receiving telephone counseling at baseline after they had been informed that the process of obtaining verbal consent was audio-taped by nurse counselors.

### Design

Local women’s organizations collaborated to launch a project to provide gender-specific smoking cessation services in 2006. The major objective was to publicize the need for cessation among female smokers, and encourage and support those who wanted to quit by providing gender-specific cessation counseling. The project had three phases. The first phase included creation of the Women Against Tobacco Taskforce (WATT). This taskforce aimed to mobilize the community to: support smoking cessation among female smokers; implement a needs assessment survey to ascertain the learning needs, knowledge, attitudes, and practice relating to tobacco control and smoking cessation; and identify those interested in joining the gender-specific smoking cessation training program. In the second phase, a smoking cessation training curriculum was developed and a workshop was held for female volunteers to equip them with knowledge and skills in smoking cessation and create a rapport with WATT members. The third phase provided a Hong Kong-based female-specific smoking cessation counseling hotline to help female smokers reduce or quit smoking, improve their health and quality of life, and reduce their morbidity and increase their lifespan.

### Details of activities

#### *Phase I:* WATT and the need assessment survey

After obtaining a list of women’s organizations in Hong Kong from the Women Commission and Hong Kong Federation of Women in 2006, all 194 organizations were formally invited for collaboration by our research team. Fourteen organizations (69 service units) agreed to form a network by joining WATT to promote smoking cessation among female smokers and increase awareness of the specific health risks of smoking among women. Eight of these 14 affiliates of WATT, agreed to participate in the needs assessment survey. An anonymous self-administered questionnaire was distributed to 771 workers and volunteers in these eight WATT member organizations.

#### Phase II: Gender-specific smoking cessation training curriculum

The results of the needs assessment survey were used to design a custom-made smoking cessation counseling training program for female volunteers and staff from the WATT in 2006. A 1-day training workshop was organized for the WATT affiliates. The workshop was implemented by nurse counselors who had more than 10 years of experience in smoking cessation counseling. The curriculum was designed to recognize the characteristics of female smokers and to instruct trainees on the psychological and behavioral therapies involved in managing female smokers. As described previously [10], throughout the training a variety of topics were covered, including assessment of smoking status and the stage of readiness to change, nicotine addiction, and the provision of brief individualized advice and motivation to promote cessation, using the ‘5 A’ model developed by the Agency for Health Care Policy and Research in the United States [11]. This model includes: (1) asking about tobacco use; (2) advising cessation; (3) assessing willingness to quit; (4) assisting in the attempt to quit; and (5) arranging follow-up. At the end of the program, the participants were capable of delivering sound cessation advice to female smokers. Upon completion of the program, a certificate of attendance was awarded to the participants and their organizations for their involvement.

#### Phase III: Gender-specific smoking cessation counseling service

The trained female volunteers and staff from the WATT were encouraged to raise awareness of the risks of smoking to females’ health and the importance of smoking cessation, provide brief cessation advice to female smokers according to their needs in their respective communities, and refer interested subjects to intensive counseling delivered by our trained and experienced smoking cessation counselors. After the baseline questionnaire was completed, a cessation counseling intervention was administered by a trained counselor according to the smoker’s readiness to quit. Since individuals differed in their stage of readiness to change their behavior, counselors set different goals and used different techniques to support the individual’s smoking cessation based on their stage of readiness to change. All counselors were female registered nurses who had attended a smoking cessation counseling program organized by the School of Nursing at the University of Hong Kong and were certified as smoking cessation counselors after passing an examination.

To publicize our gender-specific cessation counseling service to the community, we produced publicity materials for display at community organizations, hospitals, District Councils, and shopping malls. Advertisements were placed in newspapers and magazines, on the radio, and on television, and a website was created to promote the service. We also organized conferences and outreach activities, including a series of health talks and seminars.

### Measure

The anonymous self-administered questionnaire was used to identify the learning needs of WATT members in Phase I (please refer to Additional file 1). The questionnaire measured the (1) knowledge, (2) attitudes, and (3) practice of tobacco control and smoking cessation. The WATT members who took part in Phase II were asked to complete this questionnaire before, immediately after and 6 months after the training workshop.

### Statistical analyses

The data was analyzed by the Statistical Package for Social Science Software, version 20.0 for Window. Descriptive statistics were used to calculate the frequency and percentage for categorical variables, and the mean, standard deviation and range for continuous variables. Paired sample t-tests were performed to determine whether there were statistically significant changes in knowledge of and attitudes towards smoking cessation among workers and volunteers before and 6 months after the training.

## Results

### Need assessment survey

A total of 623 affiliates of the eight WATT members (response rate: 80.8%) returned the questionnaires. Of 623 respondents, 88.5% were female, 72.1% were aged 31–60 years, 61.5% were married, and 65.2% completed upper secondary education. A total of 254 respondents (52.8%) were volunteers from the women’s organizations and 99.4% had no prior training in smoking cessation interventions. Of the respondents, 201 (34.7%) were living with at least one smoker, and 23.6% had a family member(s) with a smoking-related disease. About 2.9% of the women and 4.5% of the men were current smokers.

Table 1 shows the knowledge of tobacco control among workers and volunteers of WATT. The respondents showed a moderate level of general knowledge of the effects of smoking on health (mean ± standard deviation; range) (3.9 ± 1.4; 0–7) but a gross under-estimation of the mortality risk of smoking and a common misconception of the most important way to reduce deaths. They had a greater awareness of the effects of smoking on some common diseases (2.9 ± 1.0; 0–4) than on the effects on female-specific diseases (2.9 ± 1.9; 0–6). Table 2 presents the attitudes of tobacco control among workers and volunteers of WATT. In general, they had a positive attitude towards tobacco control (3.3 ± 0.55) and to their role (3.2 ± 0.56), the staff of the women’s organizations (3.2 ± 0.61) and social workers or volunteers (2.77 ± 0.68) in smoking cessation on a 4-point scale (1 = ‘strongly disagree’ to 4 = ‘strongly agree’). Their attitudes towards female smokers were mixed, as shown by the low mean scores for both negative (2.71 ± 0.67) and positive (1.95 ± 0.55) attitudes towards female smokers. Indeed, there was no clear correlation between the positive and negative attitudes scores (*r* = 0.07, *p* = 0.08); 39.3% had advised smokers to quit in the past 12 months. Table 3 shows the practices of smoking cessation interventions among workers and volunteers of WATT. The major reason for not providing smoking cessation interventions was ‘no contact with smokers’ (46.9%). The 227 respondents who had smoking cessation counseling experience had a low level of practice (0.81 ± 0.68) on a 4-point Likert scale (0 = ‘never’ to 3 = ‘daily’) and a low level of perceived self-efficacy (2.10 ± 0.53) on a 4-point Likert scale (1 = ‘completely not’ to 4 = ‘absolutely true’) in smoking cessation counseling.

**Table 1:** Knowledge of tobacco control among workers and volunteers of WATT (*n* = 623)

	Correct responses	
	n	%^a^
*General knowledge: Effect of smoking on health*		
1. The health hazards of smoking are greater than that of outdoor air pollution. (correct)	555	89.1
2. The health hazards of smoking are less than that of drinking alcohol. (wrong)	408	65.5
3. Smoking ‘light’ cigarettes is a safe alternative to quitting. (wrong)	393	63.1
4. Among every 20 smokers, 1 of them will eventually die due to smoking. (wrong)	94	15.1
5. The health hazards of SHS are less than that of indoor air pollution. (wrong)	407	65.3
6. Preventing children and adolescents smoking is the most important in reducing smoking related deaths. (wrong)	63	10.1
7. There is no need to quit smoking as there are alternative ways to reduce or prevent smoking related illnesses. (wrong)	515	82.7
	Mean	SD
Mean number of correct responses out of 7 items (range: 0–7)	3.91	1.44
*Cigarette smoking contributes to an increased risk of the following diseases*		
Common disease		
1. Heart disease	547	89.2
2. Blood circulation problems	455	75.0
3. Airway or breathing problems	591	96.4
4. Visual problems	218	37.2
	Mean	SD
Mean number of correct responses out of 4 items (0–4)	2.91	0.97
Woman specific disease		
5. Cervical cancer	258	31.2
6. Dysmenorrhoea / Irregular menstruation cycles	158	26.8
7. Osteoporosis	364	60.5
8. Early menopause	233	39.2
9. Preterm delivery / Spontaneous abortion	496	82.1
10. Ectopic pregnancy / Stillbirth	316	53.0
	Mean	SD
Mean number of correct responses out of 6 items (range: 0–6)	2.93	1.87

^a^The total number of responses may be different for each item due to missing data

**Table 2:** Attitudes of tobacco control among workers and volunteers of WATT (*n* = 623)

	Mean	SD	Reliabilities
*Attitude to tobacco control*			0.85^a^
Tobacco advertising should be completely banned	3.30	0.64	
All forms of tobacco promotion (both direct and indirect) should be banned	3.25	0.67	
The law against smoking in public indoor areas (including restaurants, bars, karaoke) should be passed as soon as possible	3.46	0.68	
The use of ‘light’ and ‘mild’ in tobacco should be banned.	3.22	0.68	
Average score	3.31	0.55	
*Attitude to own role in tobacco control*			0.55^b^
I would initiate advising my friends to quit smoking	3.24	0.64	
It is my responsibility to remind people not to smoke in non-smoking areas	3.14	0.62	
Average score	3.19	0.56	
*Attitude to professionals’ role in tobacco control*			−0.05^b^
Staff of woman organizations should use every opportunity to help female clients stop smoking	3.20	0.61	
Advice from social workers or volunteers to help clients stop smoking is completely ineffective ^c^	2.77	0.68	
*Negative attitude to female smokers*			0.61^b^
Female smokers are more rough	2.70	0.77	
Female smokers are more bad tempered	2.72	0.73	
Average score	2.71	0.67	
*Positive attitude to female smokers*			0.72^a^
Female smokers are more mature	2.00	0.68	
Female smokers are more optimistic	1.89	0.61	
It is acceptable for women to smoke	1.97	0.77	
Average score	1.95	0.55	

^a^Cronbach alpha value

^b^Correlation coefficient

^c^The response to the item was re-coded so that the higher the score, the more positive of the attitude to the role of social workers or volunteers in tobacco control

The mean scores range from 1 ‘strongly disagree’ to 4 ‘strongly agree’

**Table 3:** Practices of smoking cessation interventions among workers and volunteers of WATT (*n* = 623)

	n	%^a^	Alpha
*Practice in smoking cessation counseling*			
Had tried to advise smokers quit in the past 12 months	227	39.3	---
Among those who did not practise smoking cessation (*n* = 335)			
*Reasons for not practising smoking cessation counseling*			
No contact with smokers	157	46.9	
Don’t have the related knowledge and skills	74	22.1	
It is not my responsibility	33	9.9	
I think smokers don’t want to be advised	79	23.6	
I think it is ineffective	89	26.6	
I don’t want to harm the relationship	55	16.4	
Others	14	4.2	
Among those who had practised smoking cessation counseling (*n* = 277)	Mean	SD	
*Practice of 5As (0 = Never to 3 = Frequently)*			0.89
Assess about level of tobacco use	1.07	1.14	
Advise to stop smoking	1.39	0.95	
Assess willingness to make a quit attempt	0.89	0.96	
Assist in quit attempt (brief advice or counseling)	1.02	0.96	
Arrange follow up contact	0.45	0.81	
Motivate clients’ intention to quit smoking	1.11	0.93	
Refer to health professionals for advice about quit smoking	0.40	0.79	
Organize seminars/health talks on tobacco and health	0.40	0.76	
Average score	0.81	0.68	
*Perceived self-efficacy in smoking cessation counseling (1 = ‘completely not’ to 4 = ‘absolutely true’)*			0.87
I believe I will be successful in helping smokers to quit	2.01	0.59	
I am confident to help smokers to quit	2.14	0.59	
I think I am competent in helping smokers stop smoking	2.16	0.60	
Average score	2.10	0.53	

^a^The total number of responses may be different for each item due to missing data

### Gender-specific smoking cessation training curriculum

#### Smoking cessation training curriculum

All of the 28 participants passed the examination and qualified to provide brief cessation advice. They were very satisfied with the arrangement of the program (3.96 ± 0.60), the variety of topics (3.92 ± 0.63), and the content (4.08 ± 0.63) on a 5-point Likert scale ranging from 1 (‘unsatisfactory’) to 5 (‘excellent’). The participants were satisfied with the topics introduced, but would have liked a longer workshop that included case studies to strengthen their counseling skills.

#### Changes in knowledge, attitudes, and the practice of tobacco control

Changes in knowledge, attitudes, and the practice of tobacco control among participants are shown in Table 4. A total of 22 participants completed the questionnaire both before and 6 months after training; 95% were female, 95% were aged ≦ 60 years, and one of them (5%) had received smoking cessation training previously. Overall, their general knowledge of the health effects of smoking improved (5.15 ± 0.7 versus 4.5 ± 1.0; *p* = 0.024; range: 0–7) while their specific knowledge of smoking-related diseases remained high. Their attitudes towards banning tobacco advertisements (3.31 ± 0.6 versus 3.43 ± 0.5) and their role in smoking cessation (3.17 ± 0.3 versus 3.30 ± 0.4) remained positive, but did not significantly change. They perceived a more negative image of female smokers at 6 months (3.22 ± 0.31 versus 2.97 ± 0.33, *p* = 0.001). Compared with the pre-training questionnaire, fewer participants (55% [12/22] versus 62.5% [15/24], *p* = 0.14) had provided smoking cessation counseling to clients during the 6 months after training, because the majority (70%) had not had contact with smokers. However, all of those who had not delivered smoking cessation interventions (100%) felt that they had gained the necessary knowledge and skills through the workshop to do so.

**Table 4:** Changes in knowledge, attitudes, and practice of smoking cessation among workers and volunteers at 6-month after training (*n* = 22)

	Baseline	6-month	
	Mean (SD)	Mean (SD)	*P* value
Knowledge			
General knowledge on health and tobacco use (range: 0–7)	4.5 ± 1.0	5.2 ± 0.7	0.02
Specific knowledge on smoking-related diseases (range: 0–10)	7.6 ± 2	7.7 ± 1.6	0.8
Attitudes ^a^			
Banning all tobacco advertisement	3.4 ± 0.5	3.3 ± 0.6	0.14
Recognition of own & professional role in smoking cessation counseling	3.3 ± 0.4	3.2 ± 0.3	0.17
Bad image of woman smokers	2.97 ± 0.3	3.2 ± 0.3	0.001
Practice in smoking cessation counseling	n (%)	n (%)	
Had practiced	15 (62.5)	12 (55.0)	-
Among those who had practiced ^b^			
Ask	0.83 (1.0)	0.92 (0.9)	
Advise	1.38 (1.1)	1.38 (0.8)	
Assess	0.92 (1.0)	0.92 (0.9)	
Assist	1.00 (1.1)	1.15 (0.9)	
Arrange	0.42 (0.9)	0.17 (0.4)	

^a^4-point Likert scale of 1 = strongly disagree to 4 = strongly agree

^b^4-point Likert scale of 0 = never to 3 = daily

#### Evaluation of the gender-specific smoking cessation counseling services

We received 895 phone calls, from which 457 eligible female smokers were recruited and received smoking cessation counseling from November 2006 to March 2012. As described in detail previously [10], about 50% of the 457 participants were recruited through mass media promotions, 30% from the Internet, and about 20% were referred by WATT organizations. A total of 204 subjects (44.6%) and 253 subjects (55.4%) selected in-person and telephone counseling, respectively at baseline. An analysis was performed to compare the smoking status, cessation history, reduced cigarette consumption, and self-efficacy against smoking at the 6-month follow-up between those who selected in-person and telephone counseling. There were no statistically significant differences in any outcomes between participants who selected in-person and telephone counseling. The seven-day point prevalence quit rate at the 6-month follow-up was 28.4% (130/457). Approximately 21.9% (100/457) had reduced their cigarette consumption by at least 50%, and the prevalence of cessation or reduction was 50.3% (230/457).

## Discussion

This study was the first to provide gender-specific individualized smoking cessation counseling services for female smokers in Hong Kong. Specifically, this study makes a contribution to the literature by presenting a program development describing the way to form a coalition, train people in smoking cessation services, and successfully launch the gender-specific smoking cessation counseling services. The overall results showed that the community-based network to promote smoking cessation was effective in helping female smokers to quit or reduce smoking, as demonstrated by a high quit rate and a significant reduction in daily cigarette consumption among participants who continued to smoke at the 6-month follow-up. Most importantly, the success of establishing the WATT provided some evidence to support the feasibility of creating such network to deliver a gender-specific smoking cessation intervention for female smokers in Hong Kong.

This study demonstrated that the trained female volunteers and staff from the WATT could successfully promote cessation among female smokers using the ‘5 A’ model. This study is consistent with a previous review study [12] that found that a brief cessation counselling session using the ‘5 A’ model, when delivered by a trained provider, could significantly increase cessation rates among pregnant smokers. Another study [13] also found that the ‘5 A’ model was feasible and effective in reducing smoking prevalence among Chilean female smokers. That study also revealed that most female smokers did significantly improved their knowledge on how to get assistance for cessation.

The counselors provide individualized advice that was tailored to female smokers over the phone to improve their self-efficacy in smoking cessation and management of withdrawal symptoms, so that they could gain confidence and increased control over their behavior. There are potential benefits of cessation telephone counseling over self-help interventions. A previous study [14] described how a quitline offered free smoking cessation services for pregnant and non-pregnant female smokers, and provided uninsured smokers services that they may not have had access to otherwise. Furthermore, enhancing self-efficacy among female smokers is crucial for successful cessation. A previous study [15] to determine characteristic that predicted smoking cessation in women after an invasive cardiovascular procedure found that self-efficacy was associated with higher commitment to smoking cessation.

### Limitations

This project was limited because only 14 out of 194 women’s organizations joined WATT and only eight of the 14 WATT members participated in our needs assessment survey. This reflects the limited support for tobacco control advocacy among local women’s organizations in the community. Women’s health should be the highest priority for women’s organizations. Staff training is the first step to building the capacity to encourage smoking cessation. More resources and efforts should be allocated to this area to create a rapport with local organizations in the community with regard to tobacco control advocacy.

### Implications for practice

This project successfully increased women’s awareness of tobacco and smoking cessation services, publicized the importance of cessation for female smokers, provided intensive gender-specific smoking cessation counseling services to female smokers, and evaluated the effectiveness of the services provided. We built a network (WATT) with local women’s organizations to work towards the goal of cessation promotion for female smokers, and demonstrated the feasibility of developing gender-specific smoking cessation services for female smokers in the community. The amendment of the Smoking (Public Health) Ordinance in 2007 included a smoking ban in all indoor restaurants and workplaces, and in many public places. The ban was extended to clubs and bars in 2009 and to over 129 open-air and two covered public transport facilities in 2010. Therefore, an increase in the demand for smoking cessation services by female smokers is expected. The Hong Kong government and others must take the initiative to evaluate similar programs, collaborate with women’s organizations, and increase resources for female-oriented smoking cessation services.

## Conclusions

The community-based network to promote smoking cessation reported in this study was effective in helping female smokers to quit or reduce smoking. The outcomes of the project demonstrate the feasibility of developing a gender-specific smoking cessation service for female smokers in the community that could serve as a community-based model for the government to establish a comprehensive smoking cessation program for female smokers in the future.

## Additional file

Additional file 1: The file is a copy of the questionnaire used in this study. The questionnaire was used to identify the learning needs of WATT members in Phase I. It measured the (1) knowledge, (2) attitudes, and (3) practice of tobacco control and smoking cessation. The WATT members who took part in Phase II were also asked to complete this questionnaire before, immediately after and 6 months after the training workshop. (DOC 168 kb)

## Abbreviations

WATT: Women against tobacco taskforce
