# Supplementary material for: Development of a community-based network to promote smoking cessation among female smokers in Hong Kong
Source: BMC Public Health. 2017 Apr 11;17:311. doi: 10.1186/s12889-017-4213-z (PMC5387243; doi:10.1186/s12889-017-4213-z)
Supplement: Additional file 1: — The file is a copy of the questionnaire used in this study. The questionnaire was used to identify the learning needs of WATT members in Phase I. It measured the (1) knowledge, (2) attitudes, and (3) practice of tobacco control and smoking cessation. The WATT members who took part in Phase II were also asked to complete this questionnaire before, immediately after and 6 months after the training workshop. (DOC 168 kb) [file 12889_2017_4213_MOESM1_ESM.doc]

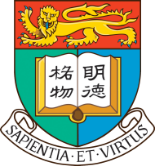


School of Nursing and School of Public Health

The University of Hong Kong

**Smoking and Health in Hong Kong Women Questionnaire**

Session 1: Please indicate how strongly you agree or disagree with each of the following statements by putting a “✓” in the appropriate box.

| Statements | Strongly Agree | Agree | Disagree | Strongly Disagree | Don’t Know |
| --- | --- | --- | --- | --- | --- |
| 1. The health hazards of smoking are greatly larger than the health hazards of air pollution. | 🞎 | 🞎 | 🞎 | 🞎 | 🞎 |
| 1. The health hazards of smoking are far fewer than the health hazards of drinking (alcohol). | 🞎 | 🞎 | 🞎 | 🞎 | 🞎 |
| 1. Smoking “light” cigarettes is a safe alternative to quitting | 🞎 | 🞎 | 🞎 | 🞎 | 🞎 |
| 1. About 1 out of 20 (i.e. 5%) smokers, if they continue to smoke, will eventually be killed by smoking | 🞎 | 🞎 | 🞎 | 🞎 | 🞎 |
| 1. The health hazards of passive smoking (or secondhand smoke) are far fewer than the health hazards of air pollution. | 🞎 | 🞎 | 🞎 | 🞎 | 🞎 |
| 1. Tobacco prevention interventions in children and adolescents is the most significant method to reduce the number of deaths caused by smoking. | 🞎 | 🞎 | 🞎 | 🞎 | 🞎 |
| 1. Tobacco cessation is not necessary for current smokers, since there are many alternative ways to prevent or reduces the risk of developing smoking-related diseases (e.g. cancer and cardiovascular disease) | 🞎 | 🞎 | 🞎 | 🞎 | 🞎 |

1. Is there a relationship between cigarette smoking and increased risk of the following diseases?

|  | Yes | No | Don’t Know |  | Yes | No | Don’t Know |
| --- | --- | --- | --- | --- | --- | --- | --- |
| 1. Cervical cancer | 🞎 | 🞎 | 🞎 | 1. Cardiovascular disease | 🞎 | 🞎 | 🞎 |
| 1. Peripheral vascular disease | 🞎 | 🞎 | 🞎 | 1. Respiratory diseases | 🞎 | 🞎 | 🞎 |
| 1. Visual impairment (e.g. Cataract) | 🞎 | 🞎 | 🞎 | 1. Dysmenorrhea/ Abnormal menstrual cycles | 🞎 | 🞎 | 🞎 |
| 1. Osteoporosis | 🞎 | 🞎 | 🞎 | 1. Early menopause | 🞎 | 🞎 | 🞎 |
| 1. Premature delivery/Spontaneous abortion | 🞎 | 🞎 | 🞎 | 1. Ectopic pregnancy/ dead fetus | 🞎 | 🞎 | 🞎 |

| 🞎 Excellent | 🞎 Good | 🞎 Regular | 🞎 Poor | 🞎 Very Poor |
| --- | --- | --- | --- | --- |

1. Overall, I perceive my knowledge in helping smokers to quit smoking is:

| 🞎 Less than 1 | 🞎 1 of them | 🞎 5 of them | 🞎 10 of them | 🞎 15 of them |
| --- | --- | --- | --- | --- |

1. Among 20 smokers, if they continue to smoke, how many of them will eventually be killed by smoking?

Session 2. Please indicate how strongly you agree or disagree with each of the following statements by putting a “✓” in the appropriate box.

| Statements | Strongly Agree | Agree | Disagree | Strongly Disagree |
| --- | --- | --- | --- | --- |
| 1. Tobacco advertising should be completely banned. | 🞎 | 🞎 | 🞎 | 🞎 |
| 1. All forms of tobacco promotion (including tobacco sponsorship) should be banned. | 🞎 | 🞎 | 🞎 | 🞎 |
| 1. Legislation to ban smoking in enclosed public places (including restaurants, bar and karaoke bar) should come into effect as soon as possible. | 🞎 | 🞎 | 🞎 | 🞎 |
| 1. Misleading terms such as “light” and “low tar” should be completely prohibited on tobacco packaging. | 🞎 | 🞎 | 🞎 | 🞎 |
| 1. I will advise my friends to stop smoking. | 🞎 | 🞎 | 🞎 | 🞎 |
| 1. I have a responsibility to reminder others not to smoke within smoke-free area. | 🞎 | 🞎 | 🞎 | 🞎 |
| 1. The staff in women organizations should take an opportunity to support female workers to stop smoking in workplaces. | 🞎 | 🞎 | 🞎 | 🞎 |
| 1. Counselling services provided by social workers or volunteers are completely ineffectual in helping their clients to quit smoking. | 🞎 | 🞎 | 🞎 | 🞎 |
| 1. Female smokers are more rude compare to female non-smokers. | 🞎 | 🞎 | 🞎 | 🞎 |
| 1. Female smokers are more emotional compare to female non-smokers. | 🞎 | 🞎 | 🞎 | 🞎 |
| 1. Female smokers are more mature compare to female non-smokers. | 🞎 | 🞎 | 🞎 | 🞎 |
| 1. Female smokers are more optimistic compare to female non-smokers. | 🞎 | 🞎 | 🞎 | 🞎 |
| 1. It is acceptable for women to smoke. | 🞎 | 🞎 | 🞎 | 🞎 |

Session 3. Past experience in providing smoking cessation counselling.

1. In the past 12 months, have you advised anyone to quit smoking?
   🞎Yes 🞎No

24a. Reason (You can tick more than one)

| 🞎 I did not get in touch with any smoker. | 🞎 I lack knowledge and skills on tobacco and health. |
| --- | --- |
| 🞎 I consider this is not my responsibility. | 🞎I consider the smokers do not want to be advised. |
| 🞎 I consider it has no effect. | 🞎I do not want to bring negative on social relationship. |
| 🞎 Other(s):______________________ |  |

24b. On average, how many clients (face-to-face) have you encountered per day?

| 🞎 None | 🞎 1-50 people | 🞎 51-100 people | 🞎 101-200 people | 🞎>200 people |
| --- | --- | --- | --- | --- |

24c. On average, what was the percentage of smoking clients you have encountered?

| 🞎 None | 🞎 1-10 % | 🞎 11-20% | 🞎 21-30% | 🞎 31-40% | 🞎 41-50% | 🞎 51% and above | Don’t Know |
| --- | --- | --- | --- | --- | --- | --- | --- |

The following are items listing specific intervention on smoking and health. Please put a “✓” in the appropriate box to indicate how often you have performed each activity in the past 12 months.

| Intervention on smoking and health | Frequently (Daily) | Occasionally (<once a day but > once a week) | Seldom (< once a week) | Never |
| --- | --- | --- | --- | --- |
| 25. Assess smoking history and status for all clients | 🞎 | 🞎 | 🞎 | 🞎 |
| 26. Advise to stop smoking | 🞎 | 🞎 | 🞎 | 🞎 |
| 27. Assess client’s readiness to quit smoking | 🞎 | 🞎 | 🞎 | 🞎 |
| 28. Help client to quit smoking (brief advise/ suggestion) | 🞎 | 🞎 | 🞎 | 🞎 |
| 29. Arrange follow-up specifically for smokers | 🞎 | 🞎 | 🞎 | 🞎 |
| 30. Encourage smoking client to quit smoking | 🞎 | 🞎 | 🞎 | 🞎 |
| 31. Refer to other health care professionals, e.g. doctor or nurse, to counsel the client about quit smoking | 🞎 | 🞎 | 🞎 | 🞎 |
| 32. Organise seminars/ health talks on tobacco and health | 🞎 | 🞎 | 🞎 | 🞎 |

Session 4. Current and future self-efficacy toward helping smoker to quit smoking.

33. In general, how do you perceive yourself in helping clients to quit smoking?

| 🞎 Very Successful | 🞎 Successful | 🞎 Unsuccessful | 🞎 Very unsuccessful |
| --- | --- | --- | --- |

34. In general, how confident are you that you can help smokers to quit smoking?

| 🞎 Very confident | 🞎 Confident | 🞎 Not very confident | 🞎 Not confident at all |
| --- | --- | --- | --- |

35. In general, how do you perceive your competence in helping clients to quit smoking?

| 🞎 Very competent | 🞎 Competent | 🞎 Not very competent | 🞎 Not competent at all |
| --- | --- | --- | --- |

Session 5. Education Needs

36. Do you want to learn and receive training regarding the following smoking and health topic(s)?

|  | Yes | No |
| --- | --- | --- |
| a. The health hazards of active smoking | 🞎 | 🞎 |
| b. The health hazards of passive smoking | 🞎 | 🞎 |
| c. The health benefits of smoking cessation | 🞎 | 🞎 |
| d. The stages of readiness to quit smoking | 🞎 | 🞎 |
| e. Nicotine addiction, tolerance, dependence and its related withdrawal symptoms | 🞎 | 🞎 |
| f. Behavioral techniques to help smokers stop smoking | 🞎 | 🞎 |
| g. Different approaches to smoking cessation intervention, e.g. interview, telephone counselling, group therapy, nicotine replacement therapy, etc. | 🞎 | 🞎 |
| h. Community resources for smoking cessation | 🞎 | 🞎 |
| i. Other(s): _________________________________ | 🞎 | 🞎 |

Session 6.

37. Which of the following item(s) currently **facilitates** you when advising **women smokers** to quit smoking in your work setting? Please put a “✓” in the appropriate box(es) (You can tick more than one).

| 1. 🞎 Quit smoking can improve the health of clients | 1. 🞎 Quit smoking is most cost-effective intervention to prevent chronic disease and cancer |
| --- | --- |
| 1. 🞎 Clients’ motivation to quit smoking | 1. 🞎 Helping clients stop smoking is part of my expected roles and responsibilities |
| 1. 🞎 Sufficient knowledge on tobacco and health | 1. 🞎 Adequate skills in smoking cessation counselling |
| 1. 🞎 Confidence in helping clients stop smoking | 1. 🞎Support from management |
| 1. 🞎 Support from colleagues | 1. 🞎 Support from health care professionals |
| 1. 🞎 Other support (e.g. family and friends) | 1. 🞎 Resources available |
| 1. 🞎 Reasonable workload | 1. 🞎 Confidence in building rapport with women smokers |
| 1. 🞎 Open to discuss the health effect of smoking for women | 1. 🞎 Other(s):__________________________ |

38. Which of the following item(s) currently **hinders** you when advising **women smokers** to quit smoking in your work setting? Please put a “✓” in the appropriate box(es) (You can tick more than one).

| 1. 🞎 Lack of time | 1. 🞎 Lack of skills (e.g. communication and counseling) |
| --- | --- |
| 1. 🞎 Lack of knowledge about how to help clients quit smoking | 1. 🞎 Lack of knowledge about tobacco and health |
| 1. 🞎 Lack of confidence in delivering smoking cessation intervention | 1. 🞎 Lack of recognition from management |
| 1. 🞎 Lack of recognition from colleagues | 1. 🞎 Lack of recognition from other parties (e.g. family and friends) |
| 1. 🞎 Lack of support from management | 1. 🞎 Lack of support from health care professionals |
| 1. 🞎 Lack of support from colleagues | 1. 🞎 Lack of support from other parties (e.g. family and friends) |
| 1. 🞎 Fear that smokers will refuse to receive smoking cessation counselling services | 1. 🞎 Believe that smoking is a kind of coping mechanism for clients under stress |
| 1. 🞎 Lack of skills in building rapport with women smokers | 1. 🞎 Uable to help smokers to resolve their life problems |
| 1. 🞎 Other(s):________________________ |  |

Session 7.

39. What is your gender? 🞎 Female 🞎 Male

40. What is your age?

🞎 <21 🞎 21-30 🞎31-40 🞎 41-50 🞎 51-60 🞎 >60

41. What is your marital status?

🞎Single 🞎 Married/De facto 🞎 Divorced/ Separated 🞎 Widowed

🞎Other (please specify):___________________

42. Do you have children?

🞎 Yes 🞎 No

43. Do you have any family member(s) who smoke(s)?

🞎 Yes 🞎 No

44. Do you have any family member(s) who died from a tobacco-related disease?

🞎 Yes 🞎 No

45. What is the highest degree or level of school you have completed?

🞎 No schooling completed 🞎 Primary School 🞎1-3 Years in secondary school

🞎 4-5 Years in secondary school 🞎 Year 6-7 in secondary school

🞎 Diploma or the equivalent 🞎 Bachelor’s degree or above
🞎Other (please specify):___________________

46. Are you currently employed?

🞎 Yes, for ___________ years 🞎 No

47. Your current position in women organization is:
🞎 Staff 🞎 Member 🞎 Volunteer 🞎Other (please specify):________________

48. Do you currently smoke/ have you been smoking before?
🞎 Never (Go to Q.52) 🞎 Quitted 🞎 Yes, I smoke occasionally. 🞎Yes, I smoke regularly.

49. How old were you when you first smoked an entire cigarette? ______________

50. How many years altogether have you been smoking daily? (i.e. smoke at least 1 more cigarette daily)

_____ Years _____ Months

51. How many cigarettes a day do you smoke?
🞎 <1 🞎 1-5 🞎 6-14 🞎 15-24 🞎 >24

52. How would you respond to secondhand smoke? (You can tick more than one)
🞎 Never exposed to secondhand smoke 🞎 No response
🞎 Use of gestures to express disaffection (e.g. fanning the smoke with hand(s), covering nose)
🞎 Move away 🞎 Ask the smoker to move away 🞎 Ask smoker to quit

53. In the past 1 month, have you ever feel discomfort due to the exposure of secondhand smoke?
🞎 Did not exposed to secondhand smoke 🞎 No discomfort
🞎 Moderate discomfort 🞎 Very discomfort 🞎 Don’t Know

54. Do you have any training in smoking cessation counseling?
🞎 Yes (please specify):___________________ 🞎 No

55. Do you wish to participate in training course about helping women to quit smoking?
🞎 Yes, please provide your contact details 🞎 No

Name: ________________ Phone Number: _________________

Email: ________________ Organization: ___________________

56. Do you have any other comment on this questionnaire?
____________________________________________________________________________________

____________________________________________________________________________________

____________________________________________________________________________________

-Thank You-
